# Delayed appearance of mature ganglia in an infant with an atypical presentation of total colonic and small bowel aganglionosis: a case report

**DOI:** 10.1186/s12887-019-1456-0

**Published:** 2019-04-05

**Authors:** Fereshteh Salimi Jazi, Julia M. Chandler, Chad M. Thorson, Tiffany J. Sinclair, Florette K. Hazard, John A. Kerner, Sanjeev Dutta, James C.Y. Dunn, Stephanie D. Chao

**Affiliations:** 1 Department of Surgery, University of Texas at Galveston, 301 University Blvd, Galveston, TX 77555 USA; 2 Division of Pediatric Surgery, Department of Surgery, Stanford University School of Medicine, 300 Pasteur Drive, Alway Building M116, MC: 5733, Stanford, CA 94305 USA; 3 Division of Pediatric Surgery, Department of Surgery, University of Miami Health System, 1120 NW 14th Street, Suite 450, Miami, FL 33136 USA; 4 Department of Pathology, Stanford University School of Medicine, 300 Pasteur Drive Rm H2110, Stanford, CA 94305 USA; 5 Department of Pediatrics – Gastroenterology, Stanford University School of Medicine, 730 Welch Rd 2nd Fl, Palo Alto, CA 94304 USA

**Keywords:** Aganglionosis, Hirschsprung’s disease, Total colonic aganglionosis, Total colonic and small bowel aganglionosis

## Abstract

**Background:**

Total colonic and small bowel aganglionosis (TCSA) occurs in less than 1% of all Hirschsprung’s disease patients. Currently, the mainstay of treatment is surgery. However, in patients with TCSA, functional outcomes are often poor. A characteristic transition zone in TCSA can be difficult to identify which may complicate surgery and may often require multiple operations.

**Case presentation:**

We present the case of a male infant who was diagnosed with biopsy-proven total colonic aganglionosis with extensive small bowel involvement as a neonate. The patient was diverted at one month of age based on leveling biopsies at 10 cm from the Ligament of Treitz. At 7 months of age, during stoma revision for a prolapsed stoma, intra-operative peristalsis was observed in nearly the entire length of the previously aganglionic bowel, and subsequent biopsies demonstrated the appearance of mature ganglion cells in a previously aganglionic segment.

**Conclusions:**

TCSA remains a major challenge for pediatric surgeons. Our case introduces new controversy to our understanding of aganglionosis. Our observations warrant further research into the possibility of post-natal ganglion maturation and encourage surgeons to consider a more conservative surgical approach.

## Background

Total colonic aganglionosis (TCA) is a rare form of Hirschsprung’s disease (HD) that affects one in every 50,000 births [1, 2]. TCA involves the entire colon and may extend up to 50 cm proximal to the ileocecal valve. Total colonic and small bowel aganglionosis (TCSA) is a more rare form involving long segments of small bowel (exceeding 50 cm) [1]. In TCSA, non-specific dilatation of small bowel loops is frequently seen. However, a characteristic transition zone can be difficult to identify [3, 4], which may complicate surgery, including the need to re-site a previously created stoma [5].

Here we present a case of apparent TCSA in which serial biopsies over the first year of life revealed de novo mature ganglion cells in previously aganglionic small bowel. This case suggests the possibility of post-natal ganglion maturation and encourages surgeons to consider a more conservative surgical approach.

## Case presentation

### Patient information

**Fig. 1:**
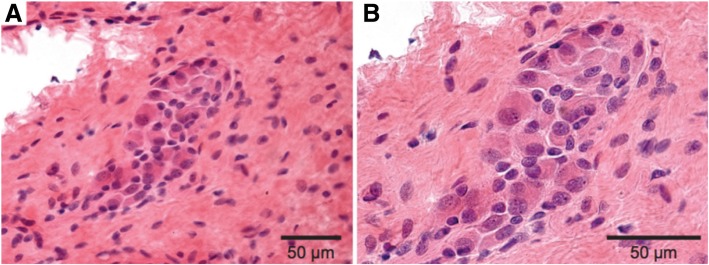
Timeline of procedures, imaging studies, and pathology findings

**Fig. 2:**
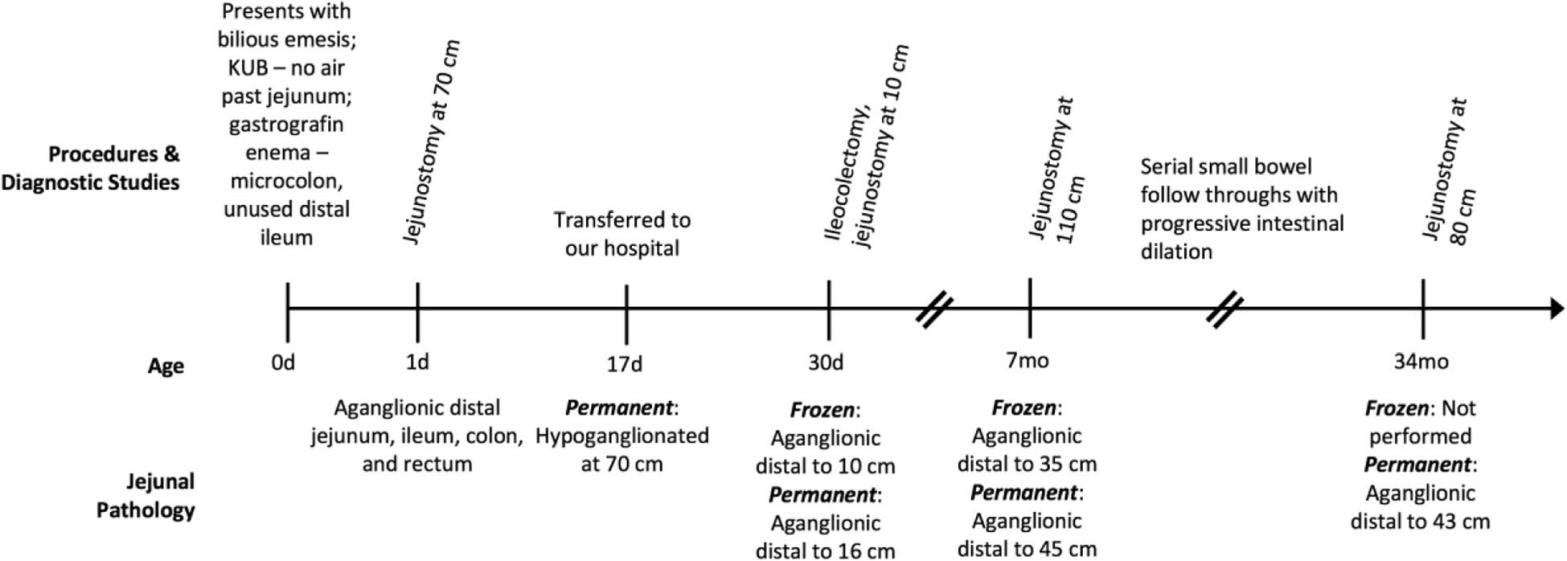
Gastrografin enema obtained at birth demonstrating a micro-colon and narrow-caliber distal ileum with absence of filling of the ileum

The patient is a term male infant born at another facility who initially presented with bilious emesis after several hours of life (Fig. 1). Pregnancy and delivery were unremarkable. Abdominal X-ray showed dilated proximal small bowel with no air in the rectum. Upper GI series demonstrated no evidence of malrotation or obstruction with a normally positioned Ligament of Treitz. No air or contrast was seen past the proximal jejunum. Follow-up gastrografin enema showed a micro-colon, unused distal ileum, and no proximal filling of the ileum (Fig. 2).

**Fig. 3:**
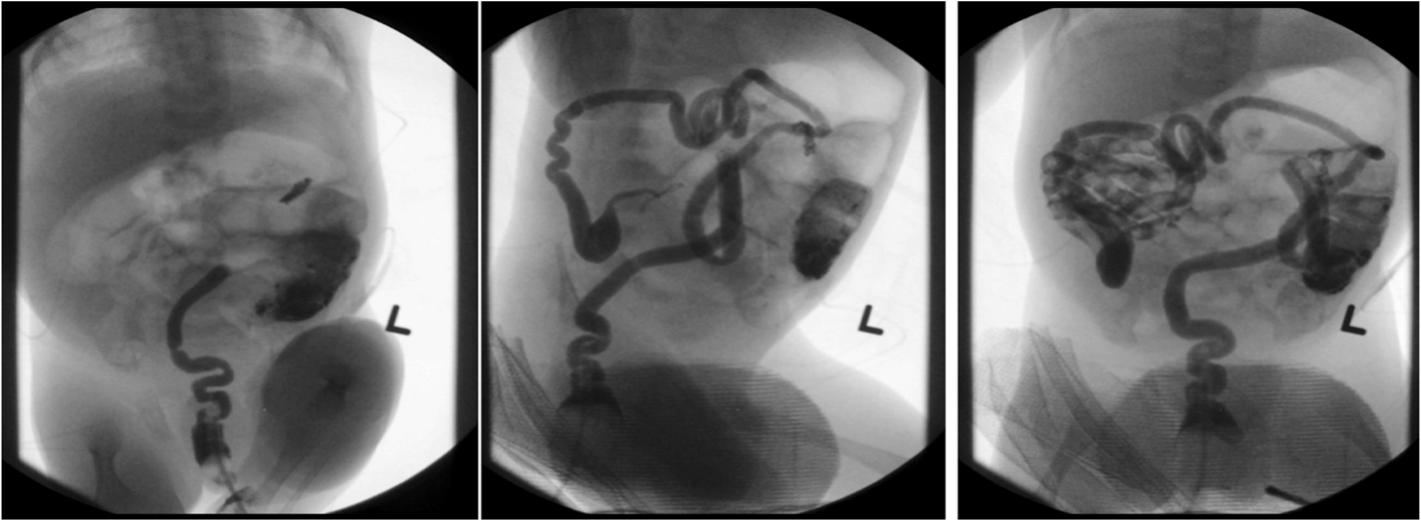
Illustration of surgeries and biopsy results. **a** – DOL1 surgery (at referring institution). **b** – DOL 30 surgery. **c** – 7 month surgery. **d** – 34 month surgery

On day of life (DOL) 1, the patient underwent urgent exploratory laparotomy for presumed jejunoileal atresia (Fig. 3a). The surgeon observed a healthy appearing micro-colon with no atresia and gradual dilatation in the mid-jejunum. Intra-operative frozen sections and permanent histopathology demonstrated no ganglion cells in three biopsies taken from the rectum, colon, or ileum; leveling biopsies of the small bowel were not performed. The patient was diagnosed with total colonic and ileal HD and underwent divided jejunostomy with mucous fistula at approximately 70 cm without bowel resection; final pathology from a biopsy of the proximal side of the jejunostomy showed ganglion cells, but no ganglion cells were seen on the distal side. Post-operatively, he continued to have bilious output through his orogastric tube and minimal stoma output.

**Fig. 4:**
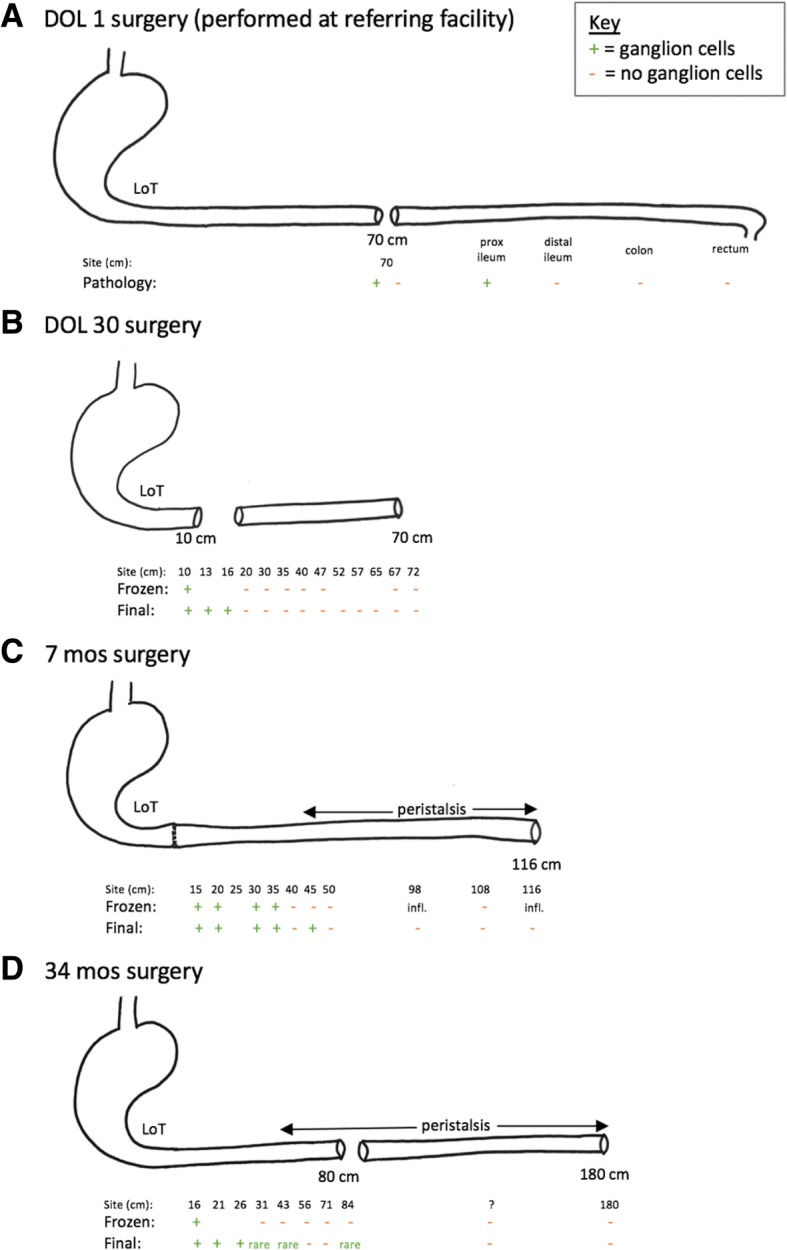
Submucosal ganglion cells in previously aganglionic bowel. **a** – Cluster of submucosal ganglion cells (H&E, 400X). **b** – Higher power magnification of submucosal ganglion cells (H&E, 600X)

### Diagnostic evaluation & therapeutic interventions

The patient was transferred to our institution on DOL 17. Evaluation for concurrent congenital anomalies and chromosomal abnormalities was negative. Our pathologists agreed that the majority of the distal bowel was aganglionic. Rare ganglion cells were noted only in the submucosa of the jejunum and proximal ileum (separated by an aganglionic segment) and thus considered hypoganglionated. A retrograde contrast study through the jejunostomy showed dilated loops of mid to distal jejunum with a transition point proximal to the jejunostomy.

On DOL 30, the infant underwent an exploratory laparotomy (Fig. 3b). Twelve seromuscular intestinal biopsies were taken, beginning distally at the jejunostomy at 70 cm and ending proximally at 10 cm distal to the Ligament of Treitz. Only the most proximal sample contained ganglion cells on frozen section. A total colectomy of the microcolon (with preservation of the rectum and distal sigmoid) and resection of the small bowel distal to the previous jejunostomy was performed. A proximal divided jejunostomy was created at 10 cm from the Ligament of Treitz. The aganglionic small bowel between the new mucous fistula and the previous stoma was not resected in favor of waiting for permanent sections.

Permanent histologic sections showed ganglionated submucosa and muscularis propria at 10, 13, and 16 cm distal to the Ligament of Treitz, but aganglionosis distal to 16 cm (Fig. 3b). Ileocolectomy specimen sections showed aganglionosis of the entire ileum and colon consistent with TCSA. The patient recovered uneventfully and his stoma began to function appropriately, but despite trials of gastrostomy tube feeding, he remained dependent on total parenteral nutrition (TPN) due to severe short gut.

At 7 months of age, he developed prolapse of the proximal mucous fistula and peri-stomal skin excoriation and underwent stoma revision (Fig. 3c). The entire length of bowel was re-examined and remeasured. The bowel had gained length since the previous operation, now measuring 116 cm. Sequential biopsies were then taken of the entire excluded segment. Normal appearing clustered ganglion cells were present on frozen section up to 35 cm from the Ligament of Treitz, without evidence of neural hypertrophy. The frozen sections were evaluated by the same pediatric pathologist. Remarkably, robust craniocaudal peristalsis was observed in the majority of the remaining (also previously aganglionic) small bowel, but evaluation of distal biopsies by frozen section demonstrated significant inflammation and could not clearly identify ganglion cells. The total bowel length had increased 1.6 fold, but the ganglionated segment appeared to have increased by at least 3.5 fold, suggesting the de novo appearance or delayed maturation of ganglion cells. Given the obvious peristalsis and ambiguity from frozen sections, the proximal jejunostomy was reanastomosed and a distal end jejunostomy was created approximately 110 cm distal to the Ligament of Treitz, where clear, repeated peristalsis was noted.

Permanent sections demonstrated ganglion cells up to 45 cm distal to the Ligament of Treitz (Fig. 4). Extensive histopathologic evaluation, including 100 consecutive sections, hematoxylin and eosin staining, and Calretinin immunohistochemistry was all consistent with TCSA. This was true even in biopsies taken from visibly peristaltic bowel, where Calretinin immunohistochemistry confirmed the absence of both submucosal ganglion cells and processes of nerve twigs within the lamina propria and muscularis mucosae. Two pediatric and two senior adult gastrointestinal pathologists independently reviewed the specimens and rendered concordant interpretations.

The patient had no significant complications for the next 27 months but did not progress significantly on his enteral feeds. He remained TPN-dependent with enteral feedings representing approximately 20% of his daily caloric intake. He also developed oral aversion to many food consistencies. At age 34 months of age, his growth followed 75th and 35th percentile for length and weight respectively. His liver function tests were essentially normal: his most recent ALT, AST, and bilirubin were 10 IU/L (range 10–175), 12 IU/L (range (17–107) and 0.1 mg/dL (range 0.1–0.5), respectively.

Although his stoma output remained around 400–500 mL/day, serial small bowel follow through examinations showed progressive intestinal dilation. He had cyclical emesis on a weekly basis. There was concern that the gradual distention was due to the aganglionic bowel causing partial obstruction which might lead to future dysfunction of ganglionic bowel.

Therefore, at 34 months of age, the patient underwent surgery for new leveling biopsies with a plan for more proximal diversion (Fig. 3d). The bowel had again lengthened since the prior surgery, now measuring 180 cm total. Once the bowel was fully mobilized, no tonically contracted segments nor a single clear transition zone were noted. Instead, there were alternating segments of dilated and non-dilated bowel throughout the abdomen corresponding more with areas of dense adhesions, rather than a transition zone. Peristalsis was noted throughout. A total of 10 leveling biopsies were taken. Given the difficulty in frozen section interpretation, the abdomen was temporarily closed to wait for permanent sections. Of note, the patient had very friable bowel at this operation.

On final pathology, leveling biopsies demonstrated single ganglion cells to very small clusters seen up to 43 cm from Ligament of Treitz. After discussion with parents and colleagues, the decision was made to create a stoma in a non-dilated portion proximal to the previous stoma but still within aganglionic bowel. The consensus was to avoid creating a stoma at 43 cm (the most distal location of documented ganglion cells), as this would likely create a high output fistula, predisposing the child to dehydration and without offering the likelihood of enteral independence.

On POD 2, the patient was taken back to the operating room. While the bowel was being delivered from the wound, nearly all of the previous biopsy sites dehisced due to poor bowel wall integrity. These were closed and, where possible, reinforced with inversion tapering enteroplasty. At approximately 80 cm, a healthy segment of non-dilated bowel was identified and a new divided jejunostomy created. The remaining bowel was preserved with a mucous fistula at the distal end to facilitate access to the defunctionalized bowel. Final pathology from the stoma specimen at 80 cm demonstrated 3–5 ganglion cells within the submucosa but none within the muscularis propria. The post-operative course was significant for the development of an enterocutaneous fistula which closed via vacuum assisted closure device on POD 7.

### Follow-up & outcomes

Ten months after the last surgery the patient is doing well with adequate weight gain. He is still TPN-dependent and is on cyclic enteral feeds with daily stoma output of 400–500 mL. Interestingly, after the last operation, he has developed an interest in eating by mouth, in contrast to his preoperative oral aversion. He has even been asking for new foods that he previously refused.

## Discussion and conclusions

TCSA occurs in less than 1% of all HD patients and may represent an entirely separate disease process from rectosigmoid HD [1, 5]. Our case introduces new controversy to our understanding of aganglionosis. We believe this is the first case of aganglionosis reported with de novo post-natal appearance of ganglion cells. Furthermore, this is the first case of witnessed propulsive peristalsis in a segment of aganglionic, normal caliber bowel. At the time of the patient’s surgeries, it appeared as though the ganglion cells were developing de novo; in retrospective review of the case, it seems possible the bowel was severely hypoganglionic with delayed maturation of ganglion cells.

The gold standard diagnostic test of HD is intestinal biopsy demonstrating aganglionosis [5, 6]. There are inherent difficulties in relying on frozen section to make definitive diagnoses in HD. Even with permanent sections, diagnosis of TCSA can be confounded by the difficulty of identifying normal intestinal plexus histologically in the small bowel compared with the colon [7]. Neural hypertrophy is absent in cases of TCA and TCSA, further complicating the identification of a potential transition zone. We identified three case reports that describe an unusual form of HD where the patients presented with multiple skip segments of aganglionosis [8–10]. No reports have identified de novo or delayed appearance of ganglion cells on serial biopsies.

The case presented here adds to the complexity in our incomplete understanding of intestinal aganglionosis and its etiology. Murine models have demonstrated long hypoganglionic segments with increased immaturity of cells [6, 11]. Watanabe et al. described hypoganglionosis as a completely different clinical entity from HD in which there is no clear identification of a transitional zone [12]. Berger et al. introduced three different types of intestinal innervation disturbances including aganglionosis (HD), hypoganglionosis, and intestinal neuronal dysplasia [13]. A transition zone with hypo- or dysganglionosis may lead gradually into normally innervated bowel [13]. It is possible this patient exhibited extreme hypoganglionosis or immature cells that matured into ganglion cells by the subsequent biopsy at 7 months of life.

An alternate explanation is that a long, extended transition zone with very rare ganglion cells escaped detection on previous leveling biopsies, or that the patient was exhibiting skip segments [9, 14]. Shimotake et al. suggested that the type of genetic mutation in HD influences the postnatal distribution and function of enteric ganglion cells. In contrast to patients with RET gene mutation, those with SOX10 mutation show a very long histologic transition extending longer than 100 cm [14]. There also exist a few studies describing acquired aganglionosis caused by inflammation and destruction of intestinal neurons due to herpes virus infection in young adults and animal models [15, 16]. Paraneoplastic syndromes and antitumor inflammatory responses have also been demonstrated to be related to ganglion cell destruction in the myenteric plexus resulting in hypoganglionosis or HD with a long segment transition zone [17]. Though none of these studies involved newborns, these mechanisms cannot be ruled out.

Contrary to the conventional understanding that aganglionosis results in a failure of relaxation in the affected segment, this patient demonstrated effective peristalsis in the aganglionic segment that was confirmed post-operatively with normal transit time on small bowel follow-through radiograph and normal stoma output. Walther et al. described a patient with TCSA who presented with dilated small bowel and visible peristalsis on X-ray despite biopsy-proven absence of ganglion cells distal to the stomach. Intraoperatively, distal small bowel peristalsis was observed; however, that patient died on DOL 80 of septicemia [18].

Currently, the mainstay of treatment for HD is surgery. However, in patients with TCSA, functional outcomes are often poor [19–21]. In patients with extensive small bowel involvement and less than 20–40 cm of ganglionic bowel, radical resection puts the patient at risk for short bowel syndrome, long term TPN dependency, and TPN-related complications such as liver failure and catheter-associated infections [5, 22]. Intestinal transplant is an emerging option, but is limited to a few centers and fraught with morbidity [23]. To decrease morbidity, partial preservation of aganglionic segments has been reported despite evidence of its association with recurrent episodes of enterocolitis [1, 22]. Others have advocated using aganglionic bowel as an intestinal patch or performing myectomy-myotomy, although these have been met with limited success and are not the standard practice at our institution [24]. Sharif et al. evaluated post-intestinal transplant outcomes of patients with TCSA with or without extensive aganglionic segment resection. Those with previous extensive resection had earlier deterioration of liver function requiring earlier transplantation and increased technical difficulty due to decreased intraabdominal space [25]. Our finding of propulsive peristalsis in apparently aganglionic, but normal caliber bowel may support the practice of preservation of aganglionic bowel in cases of TCSA. Nevertheless, the ideal operative approach remains controversial [26, 27].

TCSA remains a major challenge for pediatric surgeons. We present the first case of TCSA in which mature ganglion cells were subsequently identified in a significant portion of previously aganglionic intestine with effective peristalsis observed in the remaining aganglionic, normal-caliber small bowel. Our observations warrant further research into the possibility of post-natal ganglion maturation in severely hypoganglionic bowel and encourage surgeons to consider a more conservative surgical approach.

## Abbreviations

DOL: day of life
H&E: Hematoxylin and eosin
HD: Hirschsprung’s disease
POD: post-operative day
TCA: Total colonic aganglionosis
TCSA: Total colonic and small bowel aganglionosis
TPN: total parenteral nutrition
