# Correction to: Delayed appearance of mature ganglia in an infant with an atypical presentation of total colonic and small bowel aganglionosis: a case report

**DOI:** 10.1186/s12887-019-1507-6

**Published:** 2019-05-28

**Authors:** Fereshteh Salimi Jazi, Julia M. Chandler, Chad M. Thorson, Tiffany J. Sinclair, Florette K. Hazard, John A. Kerner, Sanjeev Dutta, James C. Y. Dunn, Stephanie D. Chao

**Affiliations:** 1 Department of Surgery, University of Texas at Galveston, 301 University Blvd, Galveston, TX 77555 USA; 2 Division of Pediatric Surgery, Department of Surgery, Stanford University School of Medicine, 300 Pasteur Drive, Always Building M116, MC: 5733, Stanford, CA 94305 USA; 3 Division of Pediatric Surgery, Department of Surgery, University of Miami Health System, 1120 NW 14th Street, Suite 450, Miami, FL 33136 USA; 4 Department of Pathology, Stanford University School of Medicine, 300 Pasteur Drive Rm H2110, Stanford, CA 94305 USA; 5 Department of Pediatrics – Gastroenterology, Stanford University School of Medicine, 730 Welch Rd 2nd Fl, Palo Alto, CA 94304 USA

**Correction to: BMC Pediatrics**

**https://doi.org/10.1186/s12887-019-1456-0**

Following publication of the original article [1], the authors reported error on the images/figures used which also resulted in un-sequential order. The updated figures and captions are provided below.

**Fig. 1:**
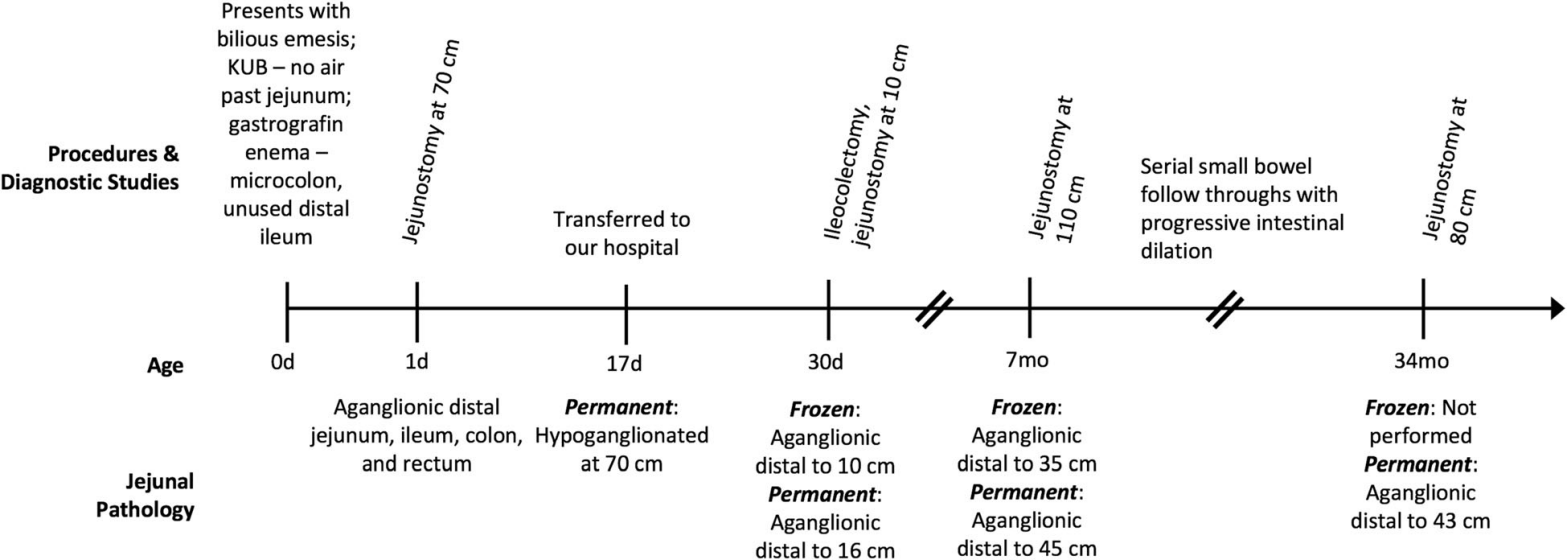
Timeline of procedures, imaging studies, and pathology findings

**Fig. 2:**
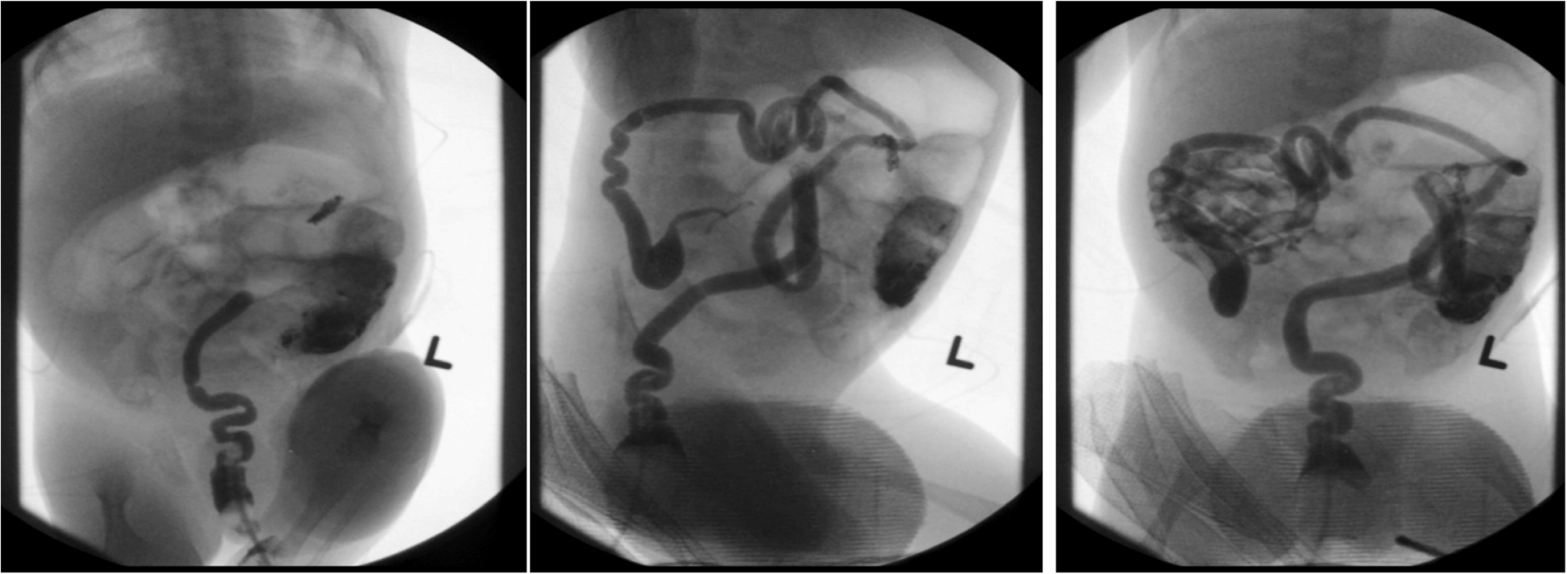
Gastrografin enema obtained at birth demonstrating a microcolon and narrow caliber distal ileum with absence of filling of the ileum

**Fig. 3:**
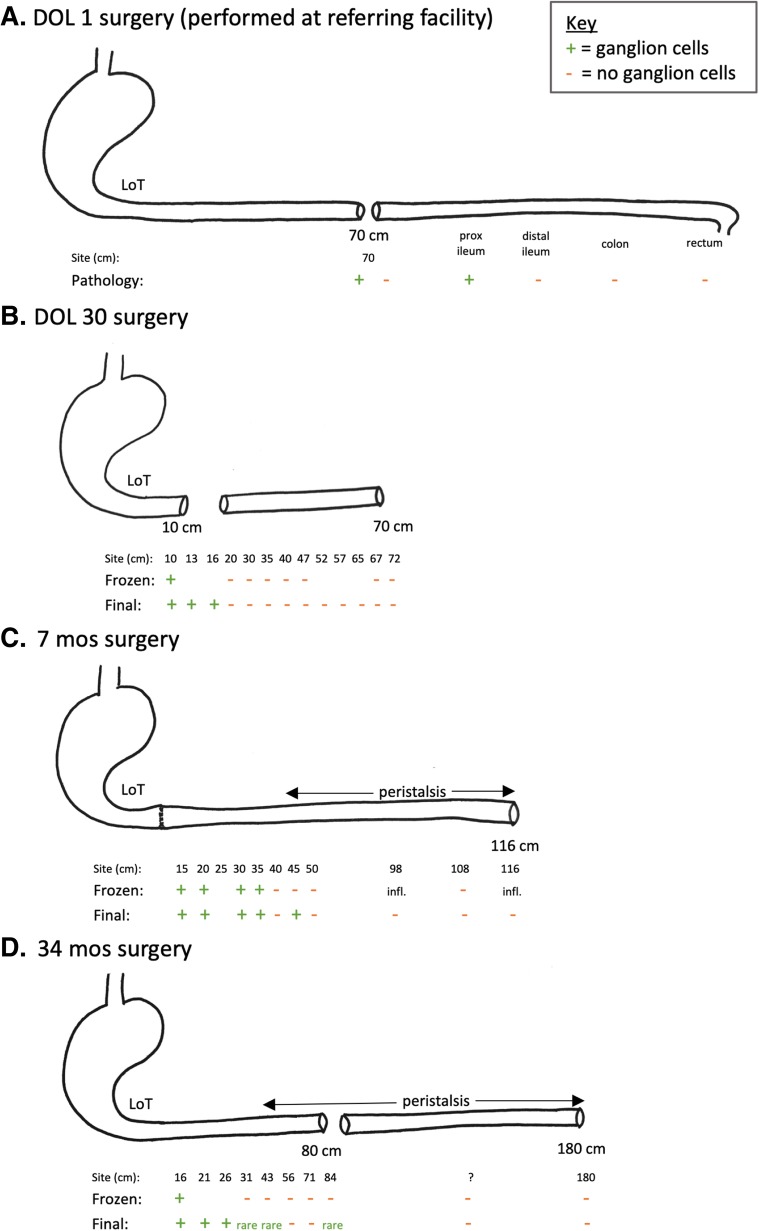
Illustration of surgeries and biopsy results. **a** DOL1 surgery (at referring institution). **b** DOL 30 surgery. **c** 7 month surgery. **d** 34 month surgery

**Fig. 4:**
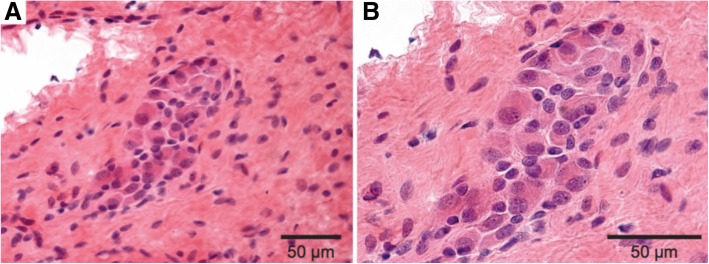
Submucosal ganglion cells in previously aganglionic bowel. **a** Cluster of submucosal ganglion cells (H&E, 400X). **b** Higher power magnification of submucosal ganglion cells (H&E, 600X)

The original article has been corrected.
